# Predictive factors affecting long-term survivorship of ASR metal-on-metal total hip arthroplasty

**DOI:** 10.1051/sicotj/2021040

**Published:** 2021-10-19

**Authors:** Sanjay Agarwala, Mayank Vijayvargiya

**Affiliations:** 1 Chief of Surgery and Director Professional Services, P.D Hinduja Hospital and Medical Research Centre 400016 Mumbai India; 2 Junior Consultant, Department of Orthopedics, P.D Hinduja Hospital and Medical Research Centre 400016 Mumbai India

**Keywords:** Articular surface replacement, Total hip arthroplasty, Revision, Adverse reaction to metal debris, Acetabular inclination

## Abstract

*Introduction*: We present the outcome of 154 ASR (Articular Surface Replacement) hips performed at the P.D Hinduja Hospital and Medical Research Centre in terms of revision rate, metal ion levels, and factors affecting survivorship. Further, determined the importance of serial metal ion estimation over single value with poorly functioning arthroplasties. *Methods*: A retrospective study of 154 ASR arthroplasties (136 patients) performed from April 2005 till March 2010 was conducted. Ninety-seven patients were available for final analysis. All patients were assessed for symptoms, radiographs, blood metal (chromium and cobalt), metal artefact reduction sequence (MARS), magnetic resonance imaging (MRI), and computerized tomography (CT). *Results*: Female gender, smaller femoral head, patients with a rising level of metal ion levels were more likely to have revision surgery. However, abnormal acetabular inclination/anteversion was not associated with the occurrence of raised metal ion levels, ARMD (adverse reaction to metal debris) or revision surgery. Patients with raised metal ion levels were more likely to have periprosthetic lucency, ARMD, and revision surgery. Median metal levels increased initially for the first three years. Still, patients who required revision surgery continued to have a metal ion rise until the point when revision surgery was performed. In contrast, patients who had a fall in metal ion levels did not require revision. *Conclusion*: A single metal ion value is less predictive of failing arthroplasties; instead, a rising trend of metal ion levels can better delineate arthroplasties which will require revision. ASR hips whose blood ion levels fell after an initial rise and showed a declining trend did well.

## Introduction

When large-diameter metal-on-metal (MoM) hip prosthesis was introduced, they offered the prospects of lower volumetric wear rates and reduced risk of dislocation [1, 2]. Further, hip resurfacing arthroplasty (HRA) could also be done, allowing for bone conservation [3]. Hence MoM prostheses gained popularity in the 1990s, especially in young and active patients. More than a million of these articulation couples were implanted worldwide [4, 5]. However, this enthusiastic acceptance of MoM articulation was sabotaged by high revisions rates.

The ASR HRA and the ASR XL for THA (ASR and ASR XL; DePuy Orthopaedics) were commercially introduced in 2004. However, it was recalled in 2010 due to unexpectedly high early revision rates reported by the Australian Joint Replacement Registry and the National Joint Registry for England Wales [6]. Multiple factors were implicated in the predisposition for implant revision, including patient-related (gender, tissue reactions, symptoms, activity level, metal hypersensitivity) or surgeon-related (component malposition, modular junctions, and component head size) [7].

An earlier revision of poorly functioning arthroplasties [8] can lead to good results, but the issue is determining this need. Poorly functioning arthroplasty has been considered as those ASR hips where either revision surgery was required, or where patients developed ARMD (adverse reaction to metal debris) or raised metal ion levels or where patients were symptomatic [8]. Various parameters, including clinic-radiological findings, metal ion levels, are being used to determine this, but the most sensitive out of them is not yet established. Studies have shown that higher metal ion levels are associated with increased wear and higher revisions [9]. Hence, one may assume a causative correlation between factors leading to higher ion levels and revision rates. Various studies have evaluated these factors; however, most of the studies are from the western world, with no studies to date have evaluated ASR outcomes in the Indian subcontinent. Thus, there is a lack of knowledge of the factors affecting ASR survivorship in Indian patients. In our study, we have analyzed the metal ion levels of patients who underwent ASR THA and have explored its association with various variables.

Studies have shown cobalt (Co), and chromium (Cr) levels correlate closely with poorly functioning arthroplasty [10]. Changing the cut-off can have different inferences; decreasing the cut-off value can increase the sensitivity and decrease the specificity for early detection of ARMD/poorly functioning arthroplasty and vice-versa. Therefore, a single metal ion value can have different inferences and outcomes. Whether serial metal ion levels will be more sensitive in recognizing this than single metal ion value is debatable. The significance of the trend of blood metal ion levels in patients with ASR implants and its relationship with clinico-radiological outcomes and revision rates have not been studied previously. The current study aimed to determine serial metal ion levels and establish their relationship with poorly functioning arthroplasties.

We present ASR hip systems’ outcomes at a tertiary care centre in Mumbai, India. Our study aimed to evaluate these patients’ outcomes in terms of revision rate, implant survival, metal ion levels, and complication rate of the ASR devices in 154 hips followed up for 15 years.

## Materials and methods

### Patients

**Figure 1:**
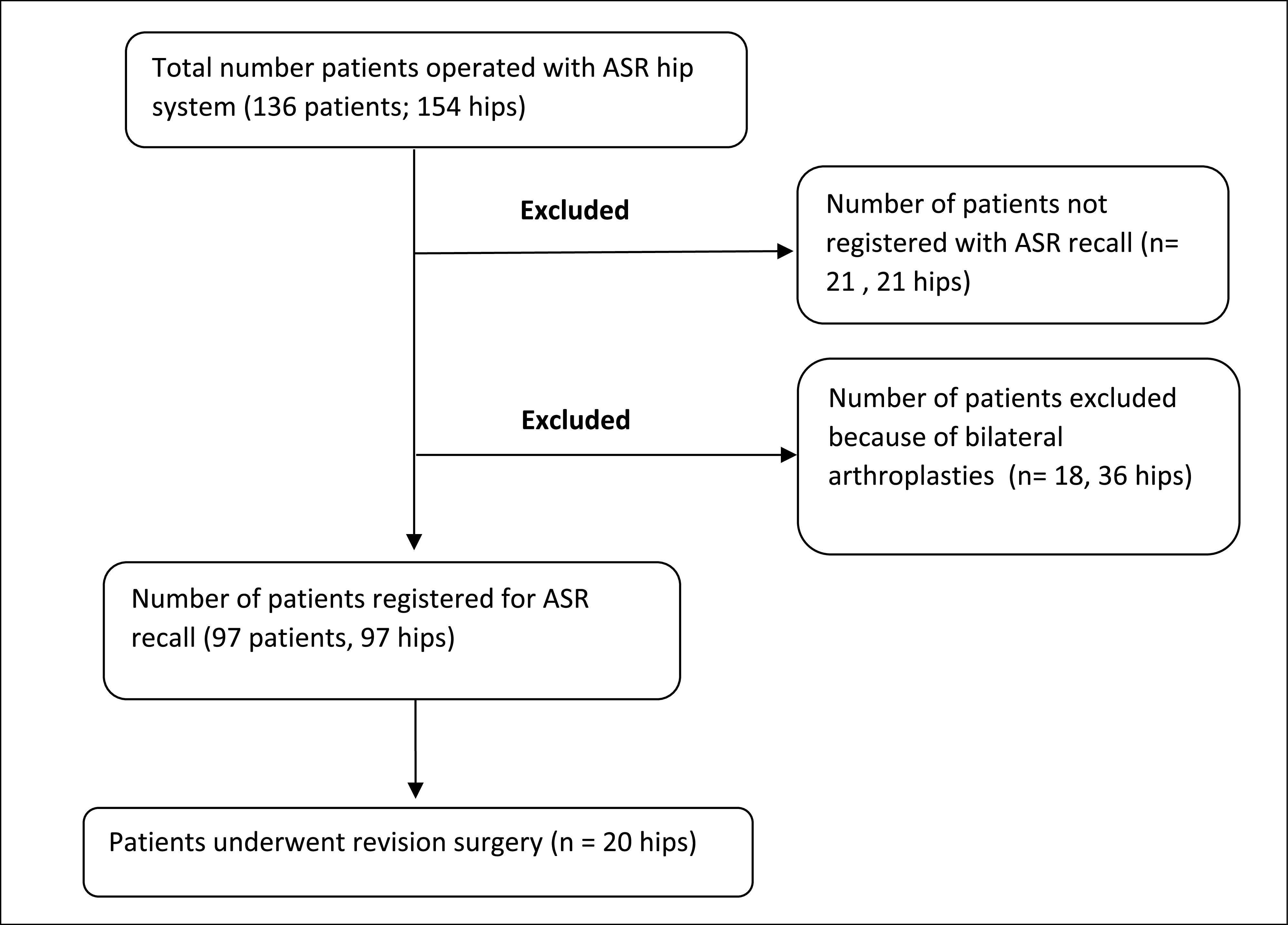
Consort Flow Diagram of the patients enrolled in this study who underwent THR using ASR system.

This retrospective study was conducted after institutional review board approval. One hundred fifty-four arthroplasties (136 patients) (80 ASR HRA and 74 ASR XL) were performed using the ASR system from April 2005 till March 2010 at our institute. All the surgeries were performed by a single senior orthopaedic surgeon using a standard anterolateral approach and the same postoperative protocol for all the patients. In August 2010, DePuy issued the voluntary recall of the ASR, and from September 2010 to February 2020, 97 patients presented to us in response to this recall (Figure 1). All the patients who had bilateral ASR hips (18 patients, 36 hips) were excluded from the study.

### Assessment

The follow-up included clinical examination, radiographs, blood metal ion (cobalt and chromium) determination, metal artefact reduction sequence (MARS), magnetic resonance imaging (MRI), and computerized tomography (CT) scan for all the patients irrespective of their symptoms. A single laboratory did whole blood chromium and cobalt metals ion levels estimation for all the patients. The blood cobalt and chromium levels ≥7 μg/L were considered elevated [11–15]. For statistical analysis, the highest value of both ions levels was used. The presence or absence of pain, mechanical, or any other symptoms were recorded at all the follow-ups.

### Image evaluation

Standard anteroposterior and lateral radiographs of both hips and MRI scans were evaluated by two independent consultant musculoskeletal radiologists blinded to the patients’ symptomatology and serum metal ion levels. The presence or absence of periprosthetic lucency was recorded, defined as a radiolucent line of >1 mm [16]. Inclination angles outside the range of 30–50 and anteversion angles outside 5–25 were designated as abnormal as determined on CT scan [17]. MARS MRI scan was done for the presence of the most considerable size of a pseudotumor/ARMD. We defined a pseudotumor is a periprosthetic collection of any size, either fluid or solid signal intensity, excluding iliopsoas bursal distention.

### Revision surgery – criteria

Revision surgery was considered if (a) presence of ARMD regardless of symptoms or blood metal ion levels, or (b) if the patient had both raised metal ion levels and symptoms, irrespective of imaging findings, or (c) symptomatic hip regardless of imaging findings or metal ion levels [18]. Patients who did not meet this criterion of revision surgery were scheduled for annual visits, and borderline cases were evaluated more frequently.

### Revision surgery – approach

The revision surgeries were conducted on 20 patients (20 joints). Delta Motion Acetabular cup comprising Ti6A14V Metal Cup and Biolox Delta Ceramic Liner was used for acetabular revision in 12 hips, and Pinnacle Marathon cup with highly crosslinked Polyethylene Acetabular Liner was used in 8 hips. Loosened femoral stems were replaced with a Corail stem. The ASR™ femoral head was removed, and Biolox Delta Ceramic Femoral Head was implanted.

### Statistical analysis

We expressed categorical variables as a number of patients and percentage of patients. Further, we compared them across the groups using Pearson’s Chi-Square test for Independence of Attributes/Fisher’s Exact Test. Continuous variables are expressed as mean, median, and range and further compared across the two groups using unpaired *t*-test if the data follows a normal distribution and Mann–Whitney test if the data does not follow a normal distribution. The statistical software SPSS version 20 was used. A *P*-value less than 0.05 is considered significant.

## Results

Ninety-seven patients (97 hips) met our inclusion criteria and were included in our evaluation. The median chromium value was 1.82 μg/L (range 0.48–45.54), and the median cobalt value was 6.54 μg/L (range 0.75–110.65). Twenty-eight patients (28.8%) had blood chromium levels above the highest safe level (>7 μg/L), and 43 (44.3%) patients had blood cobalt values above the safe threshold (>7 μg/L). The association of symptoms to the blood ions levels was not found to be statistically significant (Table 1).

**Table 1:** Association of blood Chromium and Cobalt levels with patient presenting symptoms.

	Cobalt (*n* = 97)			Chromium (*n* = 97)		
	<7 µg/L (*n* = 54)	>7 µg/L (*n* = 43)	*P* value*	<7 µg/L (*n* = 69)	>7 µg/L (*n* = 28)	*P* value*
Pain symptoms (*n* = 57)	29 (53.7%)	28 (65.1%)	0.251	41 (59.4%)	16 (57.1%)	0.511
Mechanical symptoms (*n* = 8)	5 (9.2%)	3 (6.9%)	0.680	6 (8.7%)	2 (7.1%)	0.794

* Test used Pearson’s Chi Square test for Independence of Attributes/Fisher’s Exact Test as appropriate.

Radiographic periprosthetic lucency was seen in 16.3% of 43 patients with blood cobalt value elevated above >7 μg/L, and in 21.4% of 28 patients with blood chromium elevated above >7 μg/L (Table 2). The association between periprosthetic lucency, ARMD, and elevated ion levels was found to be significant.

**Table 2:** Association of blood chromium and cobalt levels with imaging findings.

	Cobalt (*n* = 97)			Chromium (*n* = 97)		
	<7 µg/L (*n* = 54)	>7 µg/L (*n* = 43)	*P* value	<7 µg/L (*n* = 69)	>7 µg/L (*n* = 28)	*P* value*
Periprosthetic lucency on radiographs (*n* = 9)	2 (3.7%)	7 (16.3%)	0.042	3 (4.3%)	6 (21.4%)	0.036
ARMD (*n* = 61)	26 (48.1%)	35 (81.4%)	<0.001	38 (55.1%)	23 (82.1%)	0.004
Abnormal Acetabular Inclination (*n* = 24)	10 (18.5%)	14 (32.5%)	0.114	16 (23.2%)	8 (28.6%)	0.588
Abnormal Acetabular anteversion (*n* = 9)	4 (7.4%)	5 (11.6%)	0.485	6 (8.7%)	3 (10.7%)	0.765

* Test used Pearson’s Chi Square test for Independence of Attributes/Fisher’s Exact Test as appropriate.

The mean cup inclination was 44.78° (range of 31°–67.6°). Twenty-four hips (24.74%) had cup inclination outside the range of 30°–50°. Mean cup anteversion was 19.41 (6°–35°) with nine hips (9.27%) had anteversion outside the range of 5–25. The association of raised metal ion levels with the abnormal acetabular inclination and abnormal acetabular anteversion was not significant (Table 2).

The association between mean acetabular inclination and anteversion with ARMD was not significant (Table 3). Median femoral head size was smaller in patients who developed ARMD compared to patients who had not developed ARMD (*p* = 0.025). There was a strong correlation between both blood ion levels with the presence of ARMD (*p* < 0.001 for cobalt and *p* = 0.005 for chromium).

**Table 3:** Association of ARMD with imaging findings and blood ion levels.

	ARMD (*n* = 61)	No ARMD (*n* = 36)	*P* value*
Femoral head size (mm)	43 (41–59)	47 (41–55)	0.025
Mean Acetabular inclination	45.6 (31–67.6)	43.4 (34–64.6)	0.728
Mean Acetabular anteversion	20.6 (10–35)	17.4 (6–29)	0.488
Median blood cobalt levels	21.3 (4.76–110.65)	4.5 (0.75–48.67)	<0.001
Median blood chromium levels	14.6 (1.26–45.54)	1.32 (0.48–38.43)	0.005

* Unpaired *t*-test/Mann-Whitney U test depending on whether data follows normal distribution or not.

**Figure 2:**
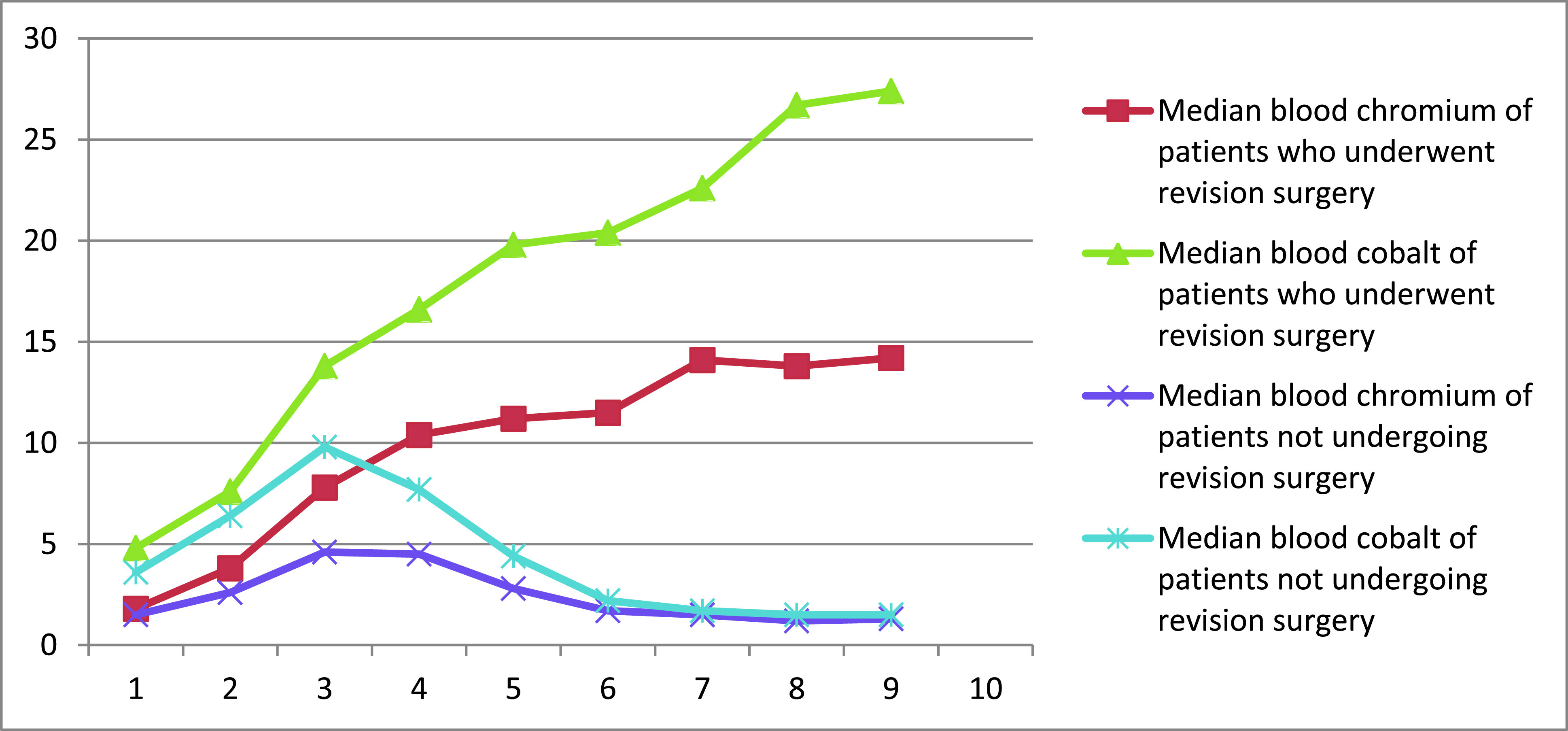
Median blood metal ion concentration trend.

The association between smaller femoral head size raised metal ion level with revision surgery was significant (Table 4). The association between mean acetabular inclination and anteversion to revision surgery was not significant. The blood metal trend is shown in Figure 2, where the metal ion level usually rises for most patients for the first three years. However, from fourth-year onwards, patients in the Revision arm (Group A) showed a continued rise in ion levels vis-à-vis patients in the Non-revision arm (Group B) for whom the metal ion levels plateau or begin to decline.

**Table 4:** Association of revision surgery with imaging findings and blood ion levels.

	Revision surgery (*n* = 20)	No revision (*n* = 77)	*P* value*
Femoral head size (mm)	43 (41–57)	49 (41–59)	0.0025
Mean Acetabular inclination	46.4 (36–67.6)	44.7 (31–64.6)	0.428
Mean Acetabular anteversion	22.3 (8–33)	18.68 (6–35)	0.149
Median blood cobalt levels	26.7 (5.78–110.65)	1.65 (0.75–35.65)	<0.001
Median blood chromium levels	13.8 (2.67–45.54)	1.46 (0.48–32.35)	0.001

* Unpaired *t* test/Mann-Whitney U test depending on whether data follows normal distribution or not.

The revision rate in our study was 16.9%. The percentage of females undergoing revision surgery was 13.4% (*n* = 13) and for males was 7.2% (*n* = 7). Revision surgery chances were 39.4% for females (13/33) and 10.9% for males (7/64). Hence, females had a four-fold higher propensity to undergo revision surgery. There was an overall revision rate of 22.6% for the ASR XL THA system and 17% for the ASR HRA. Meantime to revision surgery after primary implantation was 6.4 years (range 43–116 months). The mean follow-up in our study was 114 months (range 43–167 months).

## Discussion

Metal-on-metal bearings offered an advantage of lower dislocation rates and lower wear rates. However, concerns of increased blood metal ion, metallosis, pseudotumor formation, and early failure rates led to MOM bearings’ diminishing use. In particular, the ASR was reported to be the worst-performing, with a revision rate documented as high as 49% at six years [19]. No literature has evaluated the ASR performance in Indian patients; therefore, we have conducted this study to analyze ASR outcomes at our institute.

Our findings suggest that abnormal plasma metal ions are not associated with hip pain or mechanical hip symptoms. To our knowledge, ours is the first report of this finding from the Asian standpoint. Our results are concordance with Chang et al.’ who reported no association between revision surgery incidence and symptoms [20]. This validates the determination of metal ion levels irrespective of symptomatology since many asymptomatic patients had raised serum metal ion levels above the safe threshold.

In our study, higher metal ion levels were associated with higher ARMD prevalence, periprosthetic lucency, and revision rates. Our study results are partly corroborated by Chang et al. [20], who reported a significant correlation between periprosthetic lucency and raised serum cobalt levels. Still, the association with chromium levels was not significant. However, our results are contrary to a study by Fox et al. [14]^,^ who reported that periprosthetic lucency was not associated with higher metal ion levels.

In our study, higher metal ion levels were seen in the ASR XL THA group compared to the ASR HRA group (*p* < 0.05). This finding was seen in few other studies also [18, 19, 21]. We observed a strong association of higher metal ion levels with the prevalence of ARMD, as shown by other studies [12, 13, 20, 22–24]. However, Matharu et al. have observed that blood ion levels below implant-specific thresholds have a lower risk of ARMD development [25].

In our cohort, no association was found between abnormal acetabular inclination and anteversion with serum ion levels >7 μg/L. Few studies have found no association between abnormal acetabular inclination and raised metal ion levels [21, 26, 27]. However, some studies have reported a positive association between higher inclination angles and higher metal ion levels [22, 28–30]. We found only two studies published so far when exploring the relation of abnormal acetabular anteversion with metal ion levels [27, 29]. Langton et al. has shown that anteversion of <10° and >20° led to increased metal ion levels [29]. We have seen no association of abnormal inclination to raised metal ions levels, as reported by Parry et al. [27].

In our cohort, the suboptimal acetabular inclination was not associated with the need for revision surgery and the prevalence of ARMD. There are six studies published till now that support our finding [14, 31–35]. However, three studies published state that suboptimal acetabular cup inclination leads to a higher incidence of ARMD and higher revision rate [13, 24, 36]. Out of the four studies that evaluated the association between acetabular anteversion with failure rate or incidence of ARMD, three have found no association [31, 33, 35], and only one study has related higher failure rates with increased anteversion angles [24]. We have used CT scan to calculate acetabular inclination angles used by only one study out of the four studies that have explored this association.

MRHA guidelines suggest a cut-off value of 7 ppb as the indicator of poorly functioning arthroplasties [37]. A cut-off value of 4.9 and 4.5 was associated with poorly functioning arthroplasties as determined by Hart et al. [12] and Sidaginamale et al. [38]. Malek et al. [15] showed that when 3.5 was determined as a cut-off value, the sensitivity increased to 87%, but the specificity dropped to 27%. Thus, a single metal ion value can have different outcomes when the cut-off value is changed. Therefore, the purpose of our study was to determine serial metal ion levels and their association with poorly functioning arthroplasties.

When the median blood ion values were plotted for all the patients, from the first year to third year post-surgery, no statistically significant difference between the two groups for serum chromium and serum cobalt was seen. However, starting from year four onwards, a statistically significant difference between the two groups (*p* = 0.002) emerged. Patients in group A continued to have a metal ion level rise until the revision surgery was performed, whereas Group B patients had a fall in serum metal ion levels. McHugh et al. [39] have shown a similar trend, where both Cr and Co levels decrease in controls; however, no decrease was seen in index cases. This finding is consistent with the other study, showing a constant increase in ion levels up to four years [40]. We have also seen no statistically significant difference between the initial ion levels in both the groups; thus, it is challenging to predict patients who will require revision based on a single blood ion level determination. Thus, sequential measurement of metal ion levels may depict a pattern that may help predict implant failure early than single absolute values. One of the strengths of our study is that all the patient’s samples and follow-up samples were sent to a single laboratory to eliminate the bias, as many laboratories use different criteria for determining metal ion levels.

There were some limitations to this study. First, the number of patients lost to follow-up was high. The second limitation is the retrospective nature of the study.

## Conclusion

Smaller femoral head size, female gender, raised metal ion levels, and presence of ARMD was associated with higher revision rates. At the same time, abnormal acetabular inclination and anteversion did not correlate with failures. We also recommend routine laboratory testing for all the ASR-operated patients irrespective of their symptomatology. Since many asymptomatic patients had raised serum metal ion levels above the safe threshold. Another important implication is that a single metal ion value can be less predictive of failing arthroplasties; instead, a rising metal ion level trend can be more informative. We have seen ASR hips that did well and are still doing well; in such cases, serial metal ion levels have declined or plateaued over time. Thus, sequential measurement of metal ion levels may depict a pattern that may help predict implant failure early than single absolute values.

## Conflicts of interest

The authors received no financial support for the research, authorship, and/or publication of this article, and there are no conflicts of interest.

## Funding

This research did not receive any specific funding.

## Ethical approval

Ethical approval was not required.

## Informed consent

This article does not contain any studies involving human subjects.

## Authors contribution

Both the authors Dr Sanjay Agarwala and Dr Mayank Vijayvargiya have contributed equally to the conception, design, drafting, acquisition, analysis and interpretation of data of the study. Both the authors have read and approved the final manuscript.
